# Reconstruction of the Far-Field Pattern of Volumetric AUTs from a Reduced Set of Near-Field Samples Collected along a Planar Spiral with a Uniform Step

**DOI:** 10.3390/s21051644

**Published:** 2021-02-26

**Authors:** Francesco D’Agostino, Flaminio Ferrara, Claudio Gennarelli, Rocco Guerriero, Massimo Migliozzi, Giovanni Riccio

**Affiliations:** 1 Dipartimento di Ingegneria Industriale, Università di Salerno, Via Giovanni Paolo II, I-84084 Fisciano, Italy; fdagostino@unisa.it (F.D.); flferrara@unisa.it (F.F.); rguerriero@unisa.it (R.G.); mmigliozzi@unisa.it (M.M.); 2 Dipartimento di Ingegneria dell’Informazione ed Elettrica e Matematica Applicata, Università di Salerno, Via Giovanni Paolo II, I-84084 Fisciano, Italy; griccio@unisa.it

**Keywords:** antenna measurements, non-redundant sampling representation of electromagnetic field, uniform planar spiral scanning, near-to-far-field transformations

## Abstract

An efficient near-to-far-field transformation (NTFFT) technique, wherein the near-field (NF) measurements are acquired along a planar spiral with a uniform step to make the control of the involved positioners easier, is developed in this article. Such a technique is tailored for quasi-spherical, i.e., volumetric, antennas under test and makes use of a reduced number of NF data. An effective two-dimensional sampling interpolation algorithm, allowing the accurate reconstruction of the input NF data for the standard NTFFT with plane-rectangular scan, is obtained by setting the spiral step equal to the sample spacing required for interpolating along a radial line according to the spatial bandlimitation properties of electromagnetic fields, and by properly developing a non-redundant representation along such a spiral. Tests results are reported to demonstrate that the proposed NTFFT technique retains the same accuracy as the standard plane-rectangular one.

## 1. Introduction

The accurate characterization of an antenna is a metrological challenge whose complexity depends on the region surrounding the antenna under test (AUT), wherein the data are measured, i.e., the near-field (NF) or far-field (FF) region, the measurement environment, the facility used to collect the measurements, and the characteristics to be determined [1,2]. Direct FF measurements are the easiest ones from a computational viewpoint, but they require large outdoor ranges to allow the characterization of electrically large antennas. In any case, drawbacks related to the transportation and mounting of these AUTs make these kinds of measurements unpractical and, due to the weather conditions and the presence of electromagnetic (EM) interferences, also inaccurate. This suggests that one should perform the characterization through an indoor test range, which benefits from the fact that measurements are performed in a controlled shielded environment, i.e., the anechoic chamber. However, only NF measurements are usually allowed there, and the required FF pattern has to be properly determined by means of a near-to-FF transformation (NTFFT) technique [1,2,3,4,5,6,7]. Although the complexity grows in such a case, the NF measurements allow us to not only obtain the complete FF pattern of the AUT, but also to exploit the available information for diagnostic purposes (microwave holography). However, the accuracy of the NTFFT results is affected by several issues, such as, for instance, the truncation of the scanning area, the presence of residual reflections, and the interaction between the probe and the AUT [8].

To determine the radiated FF pattern from the measured NF data, the NTFFT techniques make use of modal expansions of the AUT field, which, depending on the type of chosen NF scanning surface, involve plane, cylindrical or spherical waves. The choice of which scanning has to be employed is dictated by the type of the antenna, the measurement requirements, and the needed analytical and mechanical complexities.

The NTFFTs with planar scans [9,10,11,12,13,14,15,16,17,18,19,20,21,22,23] represent the better choice when dealing with high gain antennas with pencil beam radiation patterns well within the solid angle identified by the AUT edges and the measurement area ones.

The standard NTFFT with plane-rectangular scanning [9,10] is the simplest one from analytical and computational viewpoints. In any case, the scanning requires a long measurement time owing to the massive amount of required NF samples, which becomes greater and greater as the working frequency and/or the scanning plane sizes increase. The employment of plane-polar [11,12,13] and bi-polar [14,15,16] scanning makes possible a scanning over a larger area as compared to that of a plane-rectangular setup for a given dimension of the measurement chamber, a finer tuning of the anechoic chamber and, involving rotational movements, a greater accuracy. However, the corresponding NTFFTs still require a massive amount of NF measurements, which leads to long scanning times.

Over the years, the scientific and industrial communities have felt the necessity to devote their efforts to find solutions capable to lower the number of the needed NF data and speed up the characterization process, without any loss in accuracy. To this end, the theoretical results on the non-redundant representations of EM fields [24,25] have been profitably exploited in [17,18,19,20,21,22,23] to massively reduce the number of needed NF data in plane-rectangular, plane-polar, and bi-polar scans, respectively.

It can be easily recognized that a time saving can be experienced not only by reducing the number of required NF samples, but also by making their acquisition faster. To this end, Rahmat-Samii et al. suggested in [26] the use of continuous and synchronized motions of the positioners of the probe and AUT. NTFFT techniques with planar spiral scanning have been developed in [27,28,29,30,31,32,33] by suitably following such a hint. In particular, the NTFFTs in [28,29,30,31,32,33] permit a greater saving time, since, by applying the non-redundant representations [24,25] and properly exploiting the unified theories of spiral scans [32,33], they employ a reduced number of NF samples and spiral turns. Two-dimensional (2-D) optimal sampling interpolation (OSI) expansions allow one to accurately recover the huge number of NF data required by the standard plane-rectangular NTFFT [9] from the non-redundant spiral samples.

The remarkable saving of measurement time is due to both the hugely reduced number of required NF samples and to how the rotary movement of the AUT positioner and the linear one of the probe positioner are combined during the acquisition on fly. In any case, such a drastic measurement time saving is obtained at the expense of a non-uniform step of the spiral [28,29,30,31]. In fact, since the spiral step is related to the sample spacing needed for the interpolation on a radial line, the distance between two consecutive intersections of the spiral with a radial line grows on increasing the distance from the center of the scanning area. Hence, the velocity of the linear positioner cannot be constant, but must vary according to a non-trivial law to correctly draw the spiral, and this, obviously, is reflected in a complex and sophisticated control system of the linear positioner.

This article’s aim is to develop an effective NTFFT technique with planar spiral scanning for volumetric AUTs, wherein the spiral step is uniform and, hence, the velocity of the linear positioner is constant. To this end, by paralleling the reasoning made in [34] with reference to the NTFFT with a uniform helicoidal scan, the AUT is considered to be enclosed in a sphere, the spiral is chosen in such a way that its step coincides with the sample spacing needed for interpolating on a radial line according to the spatial bandlimitation properties [35], and the non-redundant representation along such a spiral is properly determined. Then, a 2-D OSI algorithm is ad hoc developed to recover the input NF data for the NTFFT [9] from the spiral NF samples.

The article is organized as follows. The introductive section is devoted to briefly reviewing the state of the art and to highlighting the motivation and interest for developing a non-redundant scanning technique, wherein, to simplify the control of the involved positioners, the NF samples are collected along a planar spiral with uniform step. The non-redundant representation of the voltage over the plane from its samples gathered along the spiral and the corresponding 2-D OSI algorithm are developed in the subsequent section. The effectiveness of the so obtained NTFFT with planar spiral scan tailored to volumetric AUTs is assessed in Section 3. Concluding remarks are provided in Section 4.

## 2. Efficient Voltage Representation over a Plane from a Reduced Number of NF Spiral Samples

The spatial bandlimitation properties of EM fields [35] and the results relevant to their non-redundant representations [24] are properly exploited to develop an efficient sampling representation of the voltage gathered by the measuring probe over a plane from its samples acquired on the spiral.

**Figure 1:**
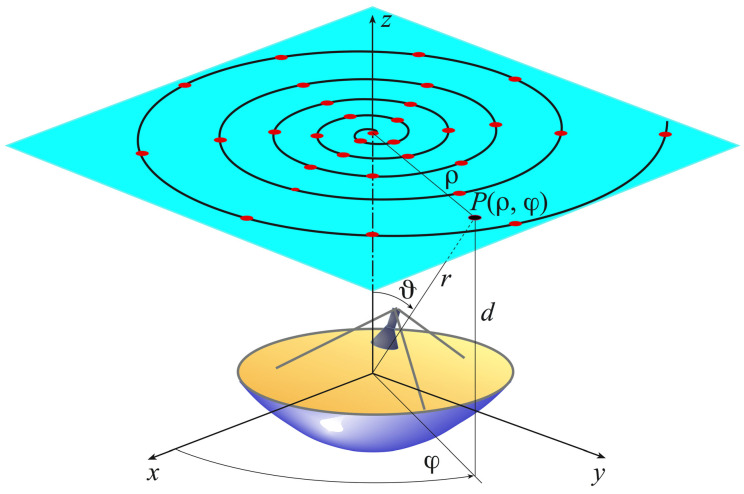
Planar spiral scanning with uniform step.

Let an electrically large volumetric, i.e., quasi-spherical, AUT be considered to be contained in a sphere of radius *a* (the smallest one enclosing it), and let an electrically small probe with a first order azimuthal dependence (first-order probe) be used to acquire the NF samples along a spiral lying on a plane placed at a distance *d* from the center *O* of the AUT. Moreover, let (*x*, *y*, *z*) be a Cartesian coordinate system centered at *O*, (r,ϑ,φ) a spherical coordinate system employed to identify an observation point *P*, and (ρ,φ) the plane-polar coordinates specifying *P* on the scanning plane (see Figure 1).

As shown in [36], the voltage *V* revealed at the terminals of the chosen probe has the same spatial bandlimitation properties of the AUT radiated field and, accordingly, the outcomes in [24] can also be applied to the measured voltage. According to [24], once the scanning spiral is represented in terms of a proper analytical parameterization $\underline{r}=\underline{r}(\eta )$ and a suitable phase factor $e^{-j\psi (\eta )}$ is singled out from the expression of *V*, it is possible to define the “reduced voltage” as

(1) $${\tilde{V}}_{\varphi ,\;\rho }(\eta )=\;V_{\varphi ,\;\rho }(\eta )\;e^{j\psi (\eta )}$$

The indices *ϕ* and *ρ* denoting the voltage of the probe and rotated probe. The so obtained reduced voltage $\tilde{V}$ is a function spatially quasi bandlimited to $W_{\eta }$ [24], and is effectively approximated by a function bandlimited to $\chi \prime W_{\eta }\;$, by choosing an excess bandwidth factor χ′, which ensures a reasonably small bandlimitation error. Note that a χ′-value slightly larger than unity is enough in the case of electrically large AUTs [24,35].

The following expressions for the optimal phase function and parameterization [24] can be used to obtain a non-redundant sampling representation of the voltage on the spiral:

(2) $$\psi \;(\eta )\;=\;\frac{\beta }{2}\int_{0}^{\sigma (Q)}\left[\underset{\underline{r}\prime }{\max}\hat{R}\cdot \;\hat{t}\;+\;\underset{\underline{r}\prime }{\min}\;\hat{R}\cdot \;\hat{t}\;\right]d\sigma$$

(3) $$\eta \;=\;\frac{\beta }{2W_{\eta }}\int_{0}^{\sigma (Q)}\left[\underset{\underline{r}\prime }{\max}\hat{R}\cdot \;\hat{t}\;-\;\underset{\underline{r}\prime }{\min}\hat{R}\cdot \;\hat{t}\;\right]d\sigma$$

where *β* is the free-space wavenumber, *σ* is the curvilinear abscissa along the spiral, $\hat{t}$ is the unit vector tangent to it at the point *Q* on the spiral, $\underline{r}\prime$ identifies the source point Q′, and $\hat{R}$ is the unit vector from Q′ to *Q*.

By denoting with *φ* the angular parameter that describes the spiral, the coordinates of *Q* can be so expressed:

(4) $$\{\begin{array}{l} x=\bar{\rho }\cos\phi \\ y=\bar{\rho }\sin\phi \\ z=d \end{array}$$

where $\bar{\rho }=\phi \;k$. Note that while the radial coordinate *ρ* is always positive, $\bar{\rho }$ can also assume negative values. Moreover, the spiral angle *φ* is continuous, whereas, according to Equation (4), the azimuthal angle *φ* has a jump discontinuity of π at the origin. The spiral step is determined by two consecutive intersections *Q*(*φ*) and *Q*(*φ* + 2π) of the scanning spiral with a given radial line. Hence, to make possible the recovery of the voltage *V* at any point *P* over the plane, such a step has to be chosen coincident with the sample spacing required, according to the spatial bandlimitation properties [35], to interpolate $\tilde{V}$ along a radial line. Accordingly,

(5) $$\Delta \rho \;=\;\frac{\pi d}{\chi \;\chi \prime \beta a}$$

*χ* being a proper oversampling factor. Being Δρ  =2π k, then k=d/(2 χ χ′βa).

**Figure 2:**
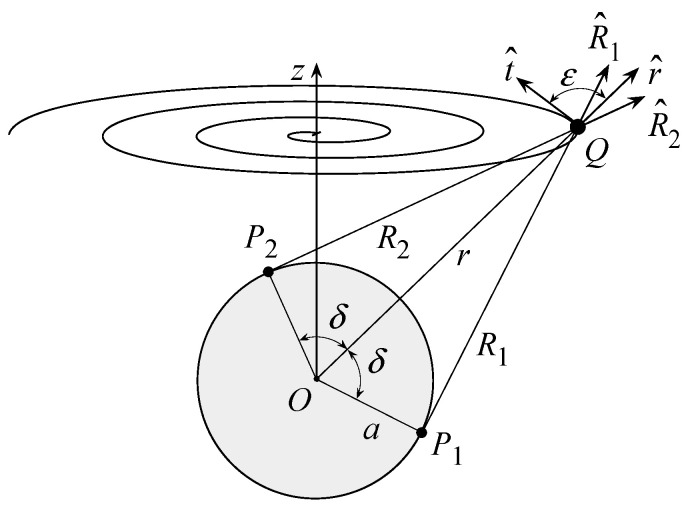
Geometry of the problem in the plane $\hat{r}$, $\hat{t}$.

Simple geometrical considerations allow one to determine the maximum and minimum values of the inner product $\hat{R}\cdot \;\hat{t}$ in Equations (2) and (3) needed to develop the non-redundant representation along the spiral. In fact, it can be easily recognized from Figure 2 that these extreme values occur at the tangency points $P_{1,2}$ of the modelling sphere, with the straight lines through the point *Q* on the spiral and lying in the plane identified by the unit vector $\hat{t}$ and that $\hat{r}$, pointing from *O* to *Q*. Indicating with ${\hat{R}}_{1,2}$ the unit vectors pointing from $P_{1,2}$ to *Q* and with $\hat{n}$ the unit vector perpendicular to $\hat{r}$ and parallel to the plane specified by $\hat{r}$ and $\hat{t}$ (see Figure 2), it results in:

(6) $$\left({\hat{R}}_{1}+\;{\hat{R}}_{2}\right)/2\;=\hat{r}\;\sin\delta =\;\hat{r}\;\sqrt{1-a^{2}/r^{2}}$$

(7) $$\left({\hat{R}}_{1}-{\hat{R}}_{2}\right)/2\;=\hat{n}\;\cos\delta \;=\hat{n}\;(a/r)$$

By substituting Equation (6) into Equation (2) and considering that $dr=\hat{r}\cdot \hat{t}\;d\sigma$, it follows:

(8) $$\psi \;=\;\beta \int_{0}^{\sigma (Q)}\;\frac{{\hat{R}}_{1}\;+\;{\hat{R}}_{2}}{2}\cdot \hat{t}\;d\sigma =\;\beta \int_{0}^{r(Q)}\sqrt{1-a^{2}/r^{2}}dr\;=\;\beta \sqrt{r^{2}-a^{2}}-\beta a\;\cos^{-1}\left(a/r\right)$$

Let us turn to the evaluation of Equation (3). To this end, denoting with *ε* the angle between $\hat{t}$ and $\hat{r}$, it results in:

(9) $$\left({\hat{R}}_{1}-{\hat{R}}_{2}\right)\cdot \hat{t}/2=\hat{t}\cdot \hat{n}\;(a/r)=(a/r)\cos\left(\varepsilon -\pi /2\right)=(a/r)\sin\varepsilon$$

In Equation (9),

(10) $$a/r\;=\;\frac{a}{\sqrt{d^{2}+k^{2}\phi^{2}}}$$

And

(11) $$\sin\varepsilon \;=\sqrt{1\;-\cos^{2}\varepsilon }\;=\sqrt{1\;-\;{\left(\hat{t}\cdot \hat{r}\;\right)}^{2}}$$

Wherein it can be easily shown that

(12) $$\hat{t}\cdot \hat{r}\;=\frac{k\phi }{\sqrt{1\;+\;\phi^{2}}\sqrt{d^{2}+k^{2}\phi^{2}}}$$

Therefore, by taking into account that from Equation (4) it follows

(13) $$d\sigma \;=\;\sqrt{{\left(dx\right)}^{2}+{\left(dy\right)}^{2}}=\;k\sqrt{1\;+\;\phi^{2}}d\phi$$

And, by choosing $W_{\eta }=\beta \;a$, Equation (3) can be rewritten as

(14) $$\eta \;=\;k\;\int_{0}^{\phi (Q)}\;\frac{\sqrt{d^{2}+\phi^{2}d^{2}+k^{2}\phi^{4}}}{d^{2}\;+\;k^{2}\phi^{2}}\;d\phi$$

Unfortunately, the integral in Equation (14) cannot be solved in a closed form and, accordingly, has to be determined numerically.

By taking into account such results, the probe voltage *V* at a point *Q* over the uniform planar spiral can be determined by using the OSI formula:

(15) $$V(\eta (Q))\;=\;e^{-j\psi (\eta (Q))}\sum_{m\;=\;m_{0}-\;p\;+1}^{m_{0}+\;p}\tilde{V}(\eta_{m})K\left(\eta -\eta_{m},\bar{\eta }\right)\sin c\left(\pi \frac{\eta -\eta_{m}}{\Delta \eta }\right)\;$$

where $\tilde{V}(\eta_{m})$ are the reduced voltage samples, $m_{0}=\;\left\lfloor \eta /\Delta \eta \right\rfloor$ is the index of the one closest to *Q*, 2*p* is the number of considered samples, ⌊·⌋ is the floor function, and

(16) $$\eta_{m}=\;m\;\Delta \eta \;=m\pi /(\chi \;\chi \prime W_{\eta })\;=m\pi /(\chi \;\chi \prime \beta a)$$

In addition, sinc(*η*) is the sin(*η*)/*η* function, and

(17) $$K(\eta ,\bar{\eta })\;=\;\frac{\cosh\left[\pi \nu p\sqrt{1-\;\left(\eta /\bar{\eta }\right)}\right]}{\cosh\left(\pi \nu p\right)}$$

With ν=(1−1/χ) and $\bar{\eta }\;=p\Delta \eta$, denotes the Knab’s sampling window function [37]. Note that when interpolating in proximity of the pole, χ′ has to be increased to avoid the bandlimitation error growing there.

Then, the reconstruction of the voltage *V* at a point *P* over the plane proceeds as follows: (a) by using Equation (15) to determine the intermediate samples on the radial line passing through *P*; (b) by interpolating these last through the OSI expansion:

(18) $$V\left(\tau (P)\right)\;=e^{-j\beta \;r(P)}\sum_{n\;=\;n_{0}-\;q\;+\;1}^{n_{0}+\;q}\tilde{V}(\tau_{n})K\left(\tau -\tau_{n},\bar{\tau }\right)\sin c\left(\pi \frac{\tau -\tau_{n}}{\Delta \tau }\right)\;$$

To finally obtain the voltage value at *P*. In this last expansion, τ=ρ/d, $\tau_{n}=\tau_{n}(\varphi )=k\varphi /d\;+n\Delta \tau =$ $\tau_{0}+n\Delta \tau$ are the normalized abscissae of the intermediate samples, $\tilde{V}(\tau_{n})=V(\tau_{n})\;e^{j\;\beta \;r\;(\tau_{n})}$ is the expression of their reduced voltages, $n_{0}\;=\;\left\lfloor (\tau \;-\tau_{0})/\Delta \tau \right\rfloor$, $\bar{\tau }=q\Delta \tau$, and the other symbols have the same meanings as in Equation (15).

By summing up, the voltage at a point *P* over the plane can be determined by proceeding as follows:

(i) The phase factor $e^{-j\psi }$ is singled out from the values of the voltage samples gathered on the spiral according to the developed representation, and the OSI Equation (15) is applied for reconstructing the involved intermediate samples;

(ii) The phase factor $e^{-j\;\beta \;r(\tau_{n})}$ is extracted from the value of the intermediate samples evaluated at the previous step, and the OSI Equation (18) is applied for evaluating the voltage value at *P*.

The 2-D OSI formula, obtained by matching the Equations (15) and (18), can be applied to reconstruct the voltages $V_{\rho }$ and $V_{\varphi }$ at the points needed by the probe-compensated NTFFT with plane-rectangular scan [9]. However, the formulas in [9] require the knowledge of $V_{y}$ and $V_{x}$ to be valid. Therefore, the probe should co-rotate in order to maintain its axes parallel to those of the AUT. The usage of a first-order probe enables a “soft” co-rotation [31], allowing us to relate the no co-rotated voltages $V_{\varphi }$ and $V_{\rho }$ to the corresponding co-rotated ones $V_{y}$ and $V_{x}$ through the relations:

(19) $$V_{y}=V_{\varphi }\;\cos\;\varphi -V_{\rho }\sin\;\varphi ;\;V_{x}=V_{\varphi }\sin\;\varphi +V_{\rho }\cos\;\varphi$$

## 3. Test Results

Some experimental results appraising the efficiency of the here developed NTFFT technique with planar spiral scanning are presented in this section. They refer to an E-plane monopulse antenna working at 10 GHz in the sum mode and made by assembling two pyramidal horns. It has been mounted in the “versatile” NF facility system available at the laboratory of antenna measurements of the University of Salerno, whose positioners (a rotating table, a vertical slide, and two turntables) are arranged in such a case to work as a plane-polar NF facility. The apertures of the considered horns, lying on the plane *z* = 0, are 8.9 cm×6.8 cm sized and their centres are 26.5 cm apart. According to the developed voltage representation, this antenna is considered to be contained in a sphere with diameter 2*a* equal to its maximum transverse dimension, i.e., 36.0 cm. The measurement plane distance *d* is 19.0 cm and the samples of the probe voltages $V_{\varphi }$ and $V_{\rho }$ are collected on a spiral covering a circular zone of radius 106.0 cm. An open-ended WR-90 rectangular waveguide, exhibiting a nearly first-order azimuthal dependence [38], has been utilized as a measurement probe. As stressed above, such a choice enables the soft co-rotation of the collected voltages according to Equation (19).

A suitable choice of the χ′-value is based on the AUT maximum transverse dimension. Given the electric maximum dimension of the considered AUT (2*a* = 12 λ, λ being the wavelength), a χ′-value equal to 1.25 ensures a bandlimitation error below −90 dB [39] and, therefore, negligible.

Then, preliminary numerical simulations have been carried out in order to conveniently choose the OSI parameters to be used in the laboratory proofs, which are able to make the reconstruction error much smaller than the measurement one, thus guaranteeing that no meaningful representation error is introduced. It is noteworthy that, once the measurement set-up characteristics have been fixed, the developed non-redundant representation depends only on the sphere modelling the AUT, and not just on the particular AUT. Accordingly, a uniform planar circular array, placed in the plane *z* = 0 and with a diameter equal to 12 λ to fit the maximum transverse dimension of the considered monopulse antenna, has been simulated. The elements of the array, elementary Huygens sources linearly polarized along the *y*-axis, are radially and azimuthally spaced by 0.45 λ and are symmetrically located with respect to the *yz*-plane. Moreover, according to the given measurement set-up characteristics, the NF samples have been simulated as acquired by a WR-90 rectangular waveguide on a spiral spanning a circular zone of radius of about 35 λ. At last, the simulations account for the choice of χ′ equal to 1.25. As already stressed, the χ′-value relevant to the representation along the spiral must be properly increased nearby the pole in order to allow the control of the bandlimitation error in that zone.

**Figure 3:**
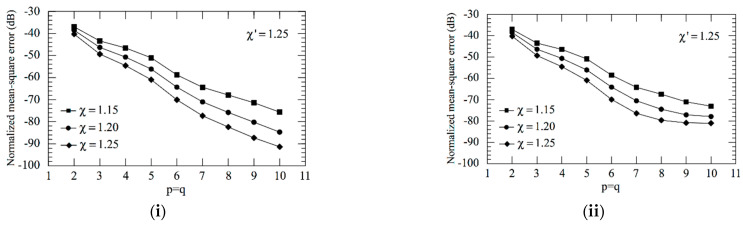
Mean-square errors in the reconstruction of $V_{\rho }$. (**i**) when reducing by a factor of 7 the sample spacing in the zones of spiral determined by the 32 samples around the pole, (**ii**) when reducing by a factor of 5 the sample spacing in the zones of spiral determined by the 22 samples around the pole.

**Figure 4:**
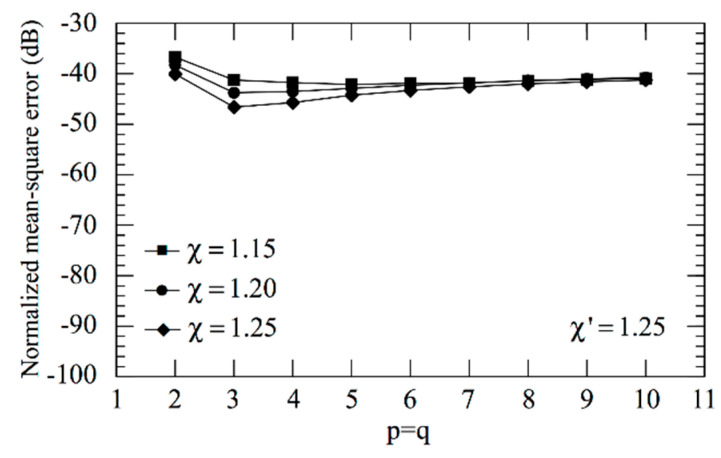
Mean-square errors in the reconstruction of $V_{\rho }$ without any increase in the χ′-value around the pole.

As an aid to an effective choice of the OSI parameters, the mean-square errors in the reconstruction of $V_{\rho }$ have been evaluated as a function of the oversampling factor *χ* and the retained sample numbers *p*, *q* and of the increase in the χ′-value around the pole. These errors have been determined by comparing the exact and recovered $V_{\rho }$ values on a close grid of the measurement zone and normalizing them to the maximum value of $V_{\rho }$ over the plane. In particular, the errors shown in Figure 3i take into account that, in the zones of the spiral determined by the 32 samples nearby the pole, the χ′-value has been augmented in such a way to reduce the sample spacing along the spiral by a factor of 7, whereas those in Figure 3ii have been obtained by reducing by a factor of 5 the sample spacing in the zones of spiral determined by the 22 samples nearby the pole. As expected, such errors decrease more and more on increasing *p*, *q* and/or *χ*, thus allowing one to choose them in such a way that the reconstruction error is remarkably lower than the measurement one. Now, set the acceptable reconstruction error threshold—a proper choice is made by determining which combination of parameters allows one to utilize the lower number of NF samples. As can be seen, *χ* = 1.20 and *p* = *q* = 8 ensure in the former case (see Figure 3i) a mean-square reconstruction error lower than the fixed threshold −75 dB, which, in the latter case (see Figure 3ii), is attained for *χ* = 1.25 and *p* = *q* = 8. Since such a threshold is obtained at the cost of a lower number of NF samples in the former case, the corresponding combination of parameters is adopted in the following. Note that such a choice assures a maximum reconstruction error of about −60 dB. For the sake of comparison, Figure 4 shows the mean-square reconstruction error in the reconstruction of $V_{\rho }$ corresponding to no increase in the χ′-value around the pole.

**Figure 5:**
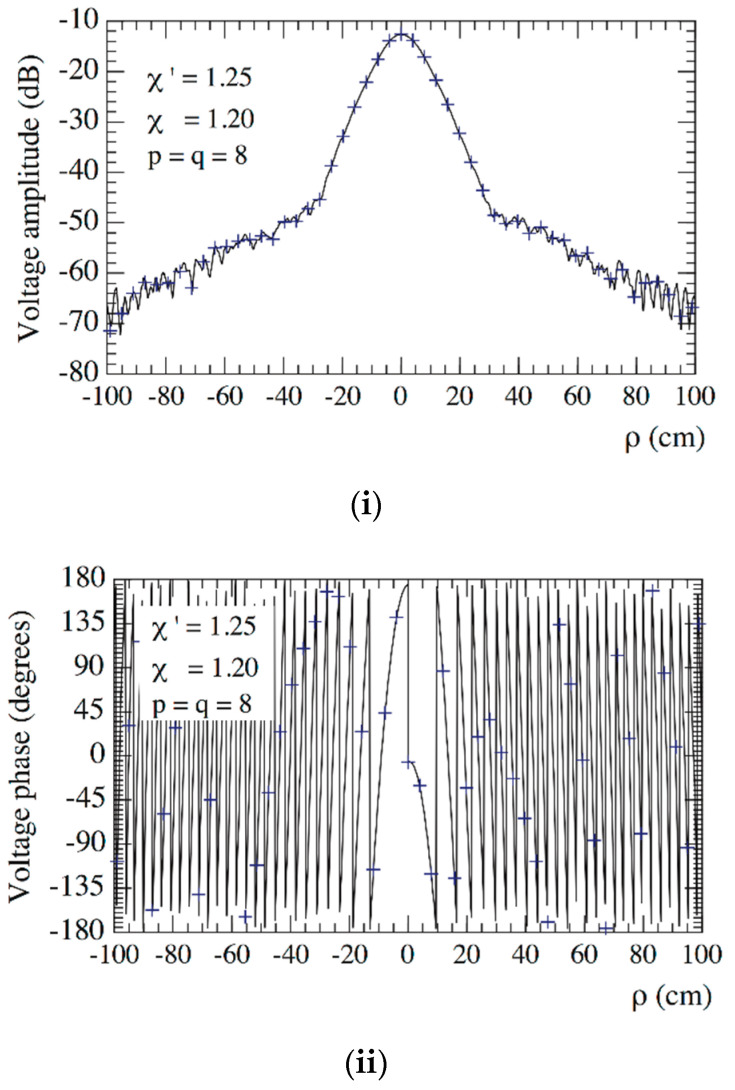
On the radial line at *φ* = 0°. ––––– reference. ++++ recovered from the planar spiral near-field (NF) measurements: (**i**) Amplitude; (**ii**) Phase.

**Figure 6:**
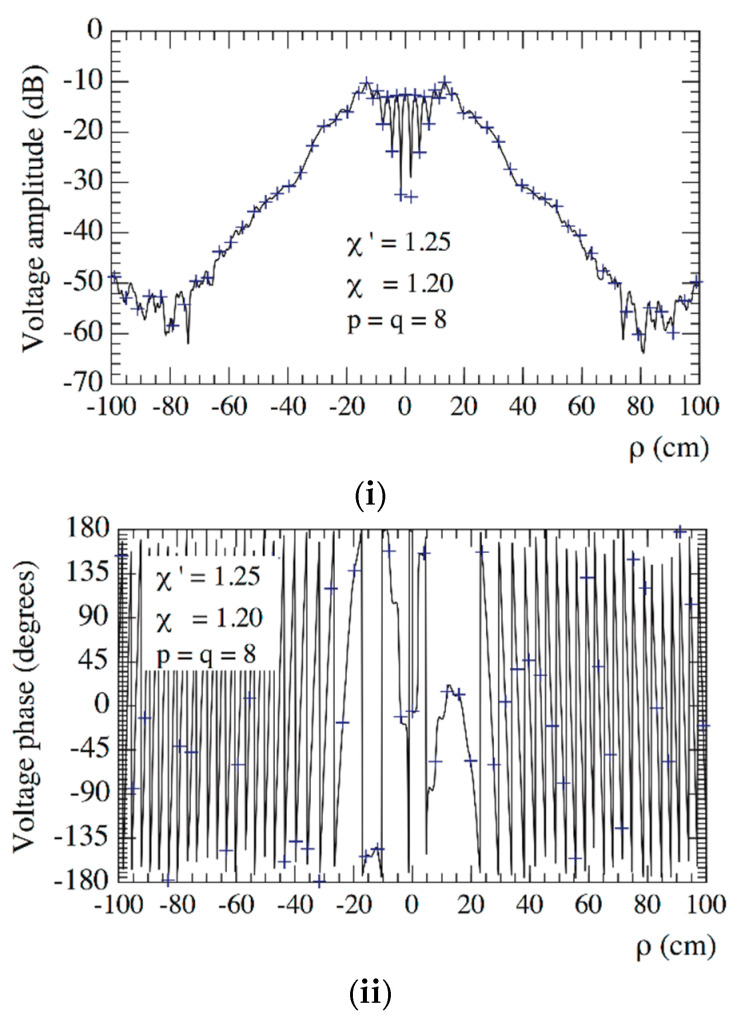
On the radial line at *φ* = 90°. ––––– reference. ++++ recovered from the planar spiral NF measurements: (**i**) Amplitude; (**ii**) Phase.

**Figure 7:**
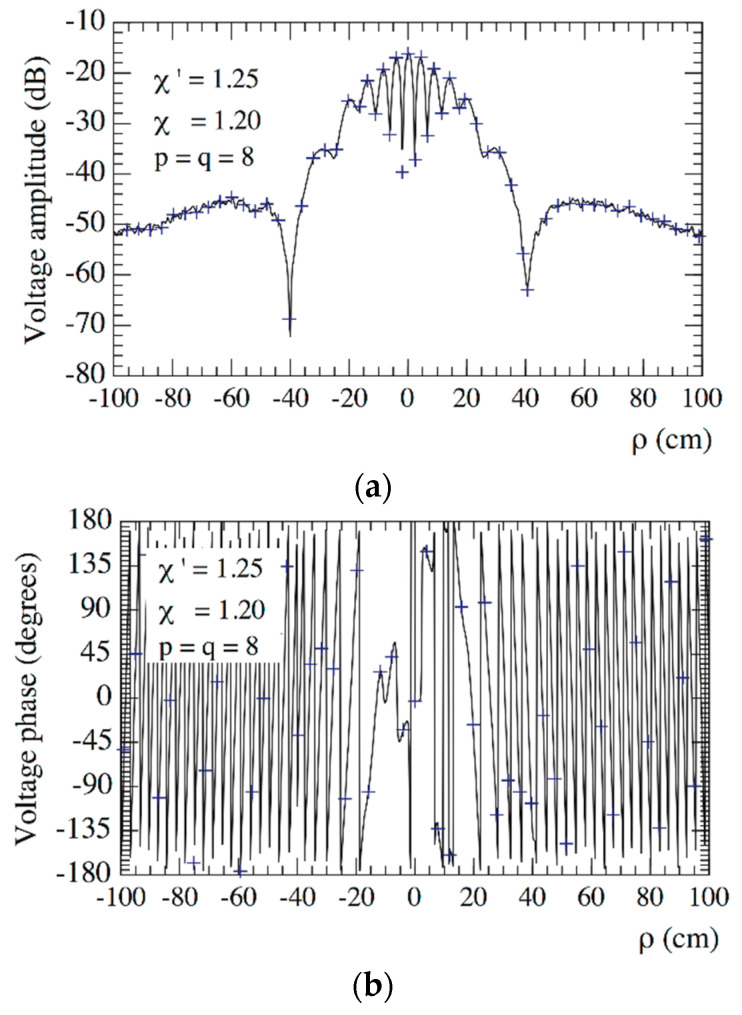
On the radial line at *φ* = 45°. ––––– reference. ++++ recovered from the planar spiral NF measurements: (**a**) Amplitude; (**b**) Phase.

Then, the so chosen parameters have been used in the experimental testing. In order to assess the precision of the 2-D OSI expansion based on these parameters, the amplitudes and phases of the recovered voltages $V_{\varphi }$ and $V_{\rho }$, relevant to the radial lines at φ=0° and φ=90°, are compared in Figure 5 and Figure 6, respectively, with the directly measured ones (references) on the same radial lines at greater resolution. For completeness, Figure 7 shows the amplitude and phase of the recovered voltage $V_{\rho }$ on the radial line at φ=45°. As can be seen, the measured voltages (solid line) and the reconstructed ones (crosses) agree very well.

**Figure 8:**
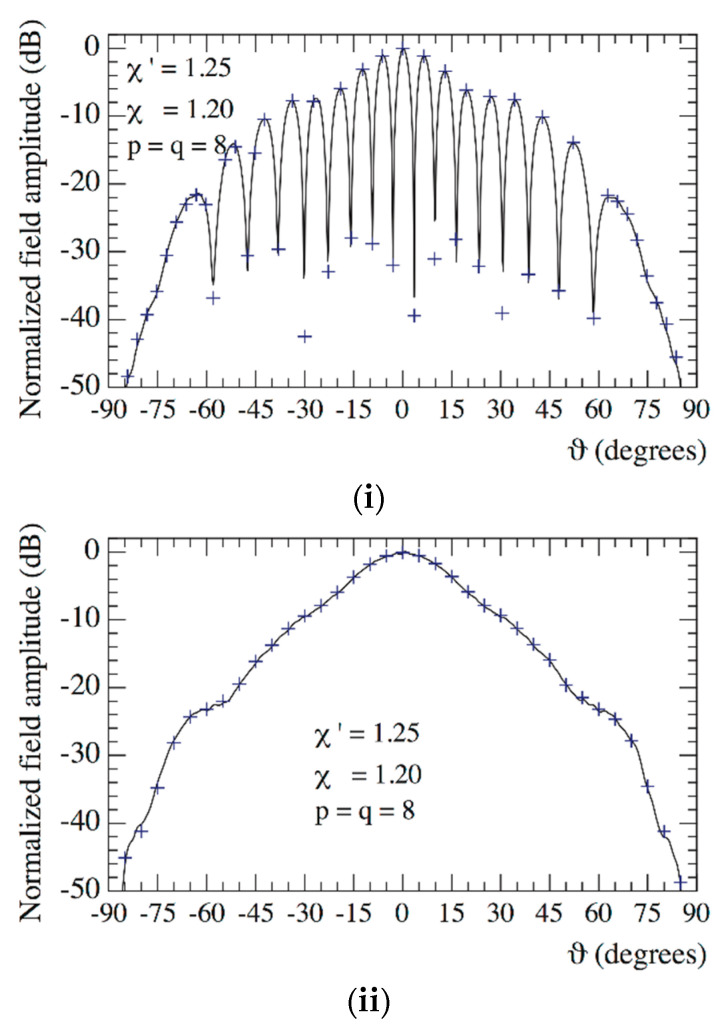
Far-field patterns. ––––– reference. ++++ reconstructed from the non-redundant samples: (**i**) E-Plane; (**ii**) H-plane.

The 2-D OSI algorithm has then been applied for the efficient reconstruction of the plane-rectangular data needed by the NTFFT [9] from the voltages $V_{\varphi }$ and $V_{\rho }$ collected along the spiral. The considered plane-rectangular sampling grid lies on a square with side 50 λ, inscribed in the measurement circle and spaced by 0.4 λ. Then, the adopted probe enables the use of Equation (19) to get, in a “soft” way, the co-rotated $V_{y}$ and $V_{x}$ voltages from the no co-rotated $V_{\varphi }$ and $V_{\rho }$ ones. Note that although the turntable between the probe and the linear positioner would have made possible a “hardware” co-rotation of the probe, its adoption would require a more complex synchronization of all the involved positioners. The so obtained principal planes patterns are compared in Figure 8 with those attained from the NF data directly acquired at the points of the considered plane-rectangular grid. For the sake of comparison, the measurement of the plane-rectangular data has been performed in such a case by acquiring the $V_{\varphi }$ and $V_{\rho }$ values through the plane-polar NF facility without the hardware co-rotation, and then applying Equation (19) to co-rotate them. As can be seen, the reconstruction process is very accurate everywhere, thus assessing the feasibility of the developed NTFFT with planar spiral scanning.

The number of NF measurements on the spiral is, for the considered example, 9812 (including 192 “extra samples” at reduced spacing around the pole). Such a number compares favorably with that (15,876) of the NF data needed by the classical plane-rectangular NTFFT [9]. It must be stressed that the developed NTFFT makes use of an increased number of NF data with respect to that (2082) required by the NTFFT in [29], exploiting the unified theory of spiral scans [32] for volumetric AUTs. In any case, since this last adopts a non-uniform step, the saving in the number of needed NF samples is obtained at the cost of a more complex control of the involved positioners.

## 4. Conclusions

An efficient NTFFT technique for volumetric AUT, wherein the number of the NF samples collected over a plane through a uniform planar spiral scan compares favorably with that needed by the classical plane-rectangular one, has been developed in this article. The proposed technique allows a significant saving of the measurement time both due to the reduction in the number of required NF data and to the way of collecting them. This spiral scanning technique, unlike those using a non-uniform step, allows one to make the synchronization of the involved positioners simpler, since the velocity of the linear one is constant during the acquisition on fly. In any case, although this is accomplished at the cost of a slight increase in both the NF data number and the acquisition time as compared to the NTFFT technique [29], the here proposed scanning technique can be easily implemented in an existing NF plane-polar facility, since it does not require any changes in the hardware controlling the positioners, but only in the software controlling them. The presented results have thoroughly demonstrated the accuracy of the sampling representation and related 2-D OSI algorithm.

## Data Availability

Data sharing is not applicable to this article.
